# Pathways to socioeconomic health differences in Armenian adolescents: The role of bullying perpetration

**DOI:** 10.1371/journal.pone.0269451

**Published:** 2022-06-03

**Authors:** Armen A. Torchyan, Hans Bosma, Inge Houkes

**Affiliations:** 1 Department of Family and Community Medicine, College of Medicine and King Khalid University Hospital, King Saud University, Riyadh, Saudi Arabia; 2 Faculty of Health, Department of Social Medicine, Care and Public Health Research Institute (CAPHRI), Medicine and Life Sciences, Maastricht University, Maastricht, The Netherlands; Gachon University Gil Medical Center, REPUBLIC OF KOREA

## Abstract

Bullying perpetration might be an alternative way of hierarchy formation among adolescents. It can potentially compensate for the negative health influences of low socioeconomic status (SES), rewarding this unwanted behavior. This study aimed to investigate the role of bullying perpetration in the relationship between SES and health among Armenian adolescents. A nationally representative sample of 3679 adolescents aged 11–15 years (mean = 13.1, standard deviation = 1.6) participated in the Health Behavior in School-aged Children 2013/14 survey in Armenia. Complex samples multiple logistic regression were used to estimate the associations between two SES measures (family socioeconomic position [SEP] and material well-being) and three health outcomes (perceived health status, psychosocial well-being, and psychosomatic symptoms). Bullying perpetration was not associated with less than good health or low psychosocial well-being (*P* > 0.05) but increased the odds of reporting high psychosomatic symptoms (*P* < 0.05). Perpetration did not change the SES-health gradient substantially. However, in stratified analyses, socioeconomic inequalities in health were consistently weaker among perpetrators. The largest observed difference was in the relationship between low family SEP and less than good health (OR = 3.60, 95% CI = 2.77–4.67 vs. OR = 1.80, 95% CI = 1.06–3.04), whereas the smallest difference was in the relationship between low family SEP and high psychosomatic symptoms (OR = 1.27, 95% CI = 1.03–1.56 vs. OR = 1.04, 95% CI = 0.61–1.77). Our findings suggest that bullying perpetration, as an alternative hierarchy, may be looked at as a compensatory but vicious strategy in the face of the negative health influences of low SES in Armenian adolescents. For high-SES adolescents, on the other hand, social, emotional, or psychological problems might contribute to bullying perpetration. Consequently, bullying prevention activities in Armenia should focus on both low and high-SES adolescents, considering SES-specific pathways and mechanisms.

## Introduction

Adolescence is a transitional period from childhood to adulthood when significant biological and social developmental processes occur [1]. During this stage, adolescents’ health is particularly vulnerable to adversities associated with low socioeconomic status (SES), which can substantially increase the risk of poor health outcomes later in life [2,3]. Multiple factors related to material, behavioral, and psychosocial pathways have been identified in the relationship between SES and adolescent health [4,5]. However, recent data show that socioeconomic inequalities in adolescent health are increasing, highlighting the need for more research and relevant policies to address this critical public health issue, especially in countries with high levels of poverty and inequality [6].

Despite the common view that young people from low-SES families are more likely to have poor health compared to their peers from high-SES families, in a study conducted among British adolescents, West [7,8] observed little variation in various health measures across SES strata in the UK. Based on his findings, he proposed the equalization in health hypothesis, which suggests that the school environment, peers, and youth culture can substantially reduce social-class differences in adolescents’ health, particularly in relation to psychosocial health outcomes [7–9]. Further studies suggested that status hierarchies present in peer-group structures, such as created by academic success, sports achievements, popularity, respect, or being a trouble-maker, can compete with SES and suppress the SES-health gradient among adolescents [9–13].

Bullying perpetration might be an alternative way of hierarchy formation among adolescents. Perhaps having no other means to improve their status, adolescents can use bullying to gain power and establish a higher position in peer-group hierarchies, pushing victims down to the bottom of the hierarchy [14–17]. Evidence suggests that the rates of mental health problems in those adolescents are low [18–20]. Moreover, in a recent study among US adolescents, bullying perpetration predicted low levels of inflammatory markers in young adulthood, suggesting long-lasting positive health effects of bullying perpetration [21]. Considering that bullying perpetration is more common among low-SES adolescents [22–24], it can, as already implied by West, potentially suppress the socioeconomic inequalities in adolescents’ health. Furthermore, bullying perpetration might alternatively be looked at as a coping and compensatory strategy for the negative influences of low SES [25,26]. This would imply a weaker relationship between low SES and poor health in perpetrators than in non-perpetrators, a pattern of associations that perhaps especially can be found in settings with high poverty levels and insufficient anti-bullying measures.

Armenia is a middle-income country, with a third of the population living in poverty [27]. Recent research indicates that SES is strongly associated with adolescent health in the country [28]. Also, bullying has been reported to be a significant public health concern in Armenia, with a lack of measures to prevent it [29], which can result in severe and long-lasting mental health consequences for victims [14]. However, the way SES, bullying, and adolescent health relate or interact in Armenia has not been studied. Therefore, in this paper, we aimed to investigate the role of bullying perpetration in the relationship between SES and health among a nationally representative sample of 3679 adolescents participating in the Health Behavior in School-aged Children (HBSC) study. Based on the above-mentioned, we propose two hypotheses. Our first hypothesis is that controlling for bullying perpetration increases the magnitude of the relationship between low SES and poor health, indicating that the influence of low SES on poor health is suppressed by bullying perpetration. The second hypothesis is that socioeconomic inequalities in health are substantially weaker in bullies, suggesting that bullying perpetration, as an alternative hierarchy, compensates for the negative health influence of low SES.

## Materials and methods

### Study design

The HBSC is a school-based survey carried out in collaboration with the World Health Organization using a standardized methodology [30]. Adolescents aged 11, 13, and 15 years (mean = 13.1, standard deviation = 1.6) were enrolled at 82 schools from all regions of Armenia, including the capital city (30.0%), towns (37.0%), and rural areas (33.1%), during November 2013 –May 2014. The list of active schools was obtained from the Ministry of Education and Science and the National Center for Educational Technologies of Armenia. The sample size was calculated using a 95% confidence interval, ± 3% precision level around 50% expected proportion, and 1.2 design factor, based on the previous data [30]. Participants were randomly selected using a clustered sampling design, where the schools and classes were the primary and secondary sampling units, respectively. The probability proportional to size (PPS) method was used to select schools, followed by a random selection of classrooms. In a classroom setting, adolescents anonymously completed the Armenian translation of the international HBSC standardized questionnaire (self-administered), which included questions on their health, health behaviors, and contextual variables [31]. Individual question descriptions and supporting scientific evidence can be found in the international HBSC protocol [30]. The HBSC study in Armenia received ethical approval before collecting data [31]. The manuscript results are based on fully anonymized secondary data, not containing student identifying information. The data supporting this study’s findings are available in the HBSC Data Management Centre [32].

### Outcome measures

The outcomes of interest for this analysis were perceived health status, psychosocial well-being, and psychosomatic symptoms. Perceived health status was self-reported by adolescents who were asked to rate their health as excellent, good, fair, or poor. The data in the latter two categories were combined and treated as less than good health (15.4% reported less than good health). Psychosocial well-being was measured with the adapted version of the Cantril Ladder [30]. Adolescents were shown a picture of a ladder and asked to indicate, in general, where on the ladder do they feel they stand at the moment. Response options ranged from 0 (worst possible life) to 10 (best possible life). The results were categorized into “low” and “high” levels of psychosocial well-being based on the median value (55.3% reported low levels). Psychosomatic symptoms included the following complaints: headache, stomachache, backache, feeling low, irritability or bad temper, feeling nervous, difficulty in getting to sleep, and feeling dizzy. The frequencies of individual psychosomatic symptoms for the preceding six months, ranging from 1 “about every day” to 5 “rarely or never” were recorded. The total symptom score was calculated by summing all eight items. The level of psychosomatic symptoms was categorized as “low” and “high” using the median value (54.1% reported low levels).

### SES measures

The family socioeconomic position (SEP) was reported by adolescents who were asked, “How well off do you think your family is?” Response categories were “quite well of,” “very well of,” “average,” “not very well of,” and “not at all well of”[30]. The lower three options were combined into a low family SEP category, which corresponds to the official poverty statistics in Armenia (approximately 30.0%) [27]. The material well-being was assessed by using the HBSC Family Affluence Scale (FAS) with four items, including the number of cars (3 categories) and computers (4) owned by the family, having their own bedroom (2), and spending the holidays abroad (4) [30]. The median value of the FAS score was used to classify the material well-being into “low” and “high” categories.

### Bullying

Before asking questions on bullying, students were presented with its definition based on the Olweus bullying questionnaire [33]. Then the students were asked two separate questions to determine the frequency of bullying perpetration and victimization: 1) “How often have you taken part in bullying another student(s) at school in the past couple of months?” 2) “How often have you been bullied at school in the past couple of months?” The answer options ranged from none to several times a week [30], and were classified into “No” or “Yes,” accordingly.

### Statistical analysis

The characteristics of adolescents were described using frequencies and percentages. A design-adjusted Chi-square test was used to test the relationship between adolescent characteristics and bullying perpetration, as well as bullying perpetration/victimization and health. Complex samples multiple logistic regression was used to estimate the associations between the SES measures and adolescents’ health outcomes. Separate regression models were estimated for each of the two SES measures and three health outcomes. Model 1 (adjusted for age, sex, and bullying victimization) was compared to Model 2 (adjusted for the variables in Model 1 and bullying perpetration) to check for possible suppression of the SES-health gradient by bullying perpetration. Subsequently, Model 1 was stratified by bullying perpetration to check whether socioeconomic inequalities in health are weaker in perpetrators compared to non-perpetrators. Two-way interactions (between SES and bullying perpetration, as well as bullying perpetration and bullying victimization) were assessed by adding product terms to the models. Further, the interaction models were stratified by sex to compare the results between boys and girls. Three-way interactions between sex, SES-measures, and bullying perpetration on health were tested by adding all pairwise product terms, as well as a product term for all three covariates to the model, after the main effects. Variables were tested for multicollinearity with variance inflation factors. *P*-values less than 0.05 were regarded as statistically significant. Data were analyzed using IBM SPSS Statistics for Windows, Version 21.0 (IBM Corp., Armonk, NY, USA).

## Results

In this study, 13.6% of students reported at least one episode of bullying perpetration in the past couple of months. The majority of perpetrators were boys (72.9%), younger than 15 years of age (80.5%), and were more likely to be bullied themselves compared to non-perpetrators (29.1% vs. 5.3%, *P* < 0.001). However, there was no statistically significant relationship (*P* > 0.05) between bullying perpetration and the SES measures (Table 1). Bullying perpetration was not associated with less than good health (OR = 1.14, 95% CI = 0.88–1.47) or low psychosocial well-being (OR = 0.96, 95% CI = 0.76–1.20) but increased the likelihood of reporting high levels of psychosomatic symptoms (OR = 2.19, 95% CI = 1.71–2.81). Bullying victimization was consistently associated with increased odds of poor health outcomes (not tabulated). The effect of bullying perpetration did not depend on victim status (P-interaction > 0.05).

**Table 1:** Frequencies (percentages) of adolescent characteristics by bullying perpetration (n = 3679 participants^a^).

Factors	No. of participants	Bullying perpetration		
		No	Yes	*P*-value
Sex				
Boys	1759	1296 (43.2)	345 (72.9)	**< 0.001**
Girls	1920	1707 (56.8)	128 (27.1)	
Age in years				
11	1471	1187 (39.5)	202 (42.7)	**0.001**
13	1163	940 (31.3)	179 (37.8)	
15	1044	875 (29.1)	92 (19.5)	
Family SEP				
High	2425	1997 (70.5)	310 (70.3)	0.946
Low	1006	837 (29.5)	131 (29.7)	
Material well-being				
High	1631	1340 (48.1)	228 (53.4)	0.056
Low	1732	1443 (51.9)	199 (46.6)	
Bullying victimization				
No	3104	2725 (94.7)	314 (70.9)	**< 0.001**
Yes	298	152 (5.3)	129 (29.1)	

SEP, socioeconomic position; Bold values denote statistical significance (*P* < 0.05).

^a^Due to missing values, not all numbers sum up to 3679.

In Model 1, adjusted for age, sex, and bullying victimization, both low family SEP and low material well-being were strongly associated with poor health outcomes, except for the relationship between low material well-being and high psychosomatic symptoms (Table 2). The relationship between bullying perpetration and adolescent health were non-significant (*P* > 0.05) for less than good health and low psychosocial well-being but remained significant (*P* < 0.05) for high psychosomatic symptoms. Bullying victimization significantly (*P* < 0.05) increased the likelihood of reporting poor health outcomes (not tabulated).

**Table 2:** Odds ratios (95% confidence interval) of poor health outcomes by two SES measures, adjusted for age, sex, and bullying victimization, without (Model 1) and with (Model 2) controlling for bullying perpetration.

Pathway	Model 1	Model 2
	OR (95% CI)	OR (95% CI)
**Less than good health**		
** Low family SEP**	**3.18 (2.51–4.03)**	**3.27 (2.58–4.14)**
** Low material well-being**	**1.68 (1.38–2.06)**	**1.69 (1.38–2.06)**
**Low psychosocial well-being**		
** Low family SEP**	**2.51 (2.04–3.10)**	**2.55 (2.06–3.15)**
** Low material well-being**	**1.49 (1.26–1.76)**	**1.50 (1.26–1.77)**
**High psychosomatic symptoms**		
** Low family SEP**	**1.24 (1.03–1.50)**	**1.24 (1.02–1.50)**
** Low material well-being**	0.93 (0.79–1.10)	0.95 (0.80–1.14)

SEP, socioeconomic position; Bold values denote statistical significance (*P* < 0.05).

Adding bullying perpetration to Model 1 did not change the SES-health gradient substantially (Table 2). However, in stratified analyses, the SES-health relationships were consistently weaker among perpetrators than non-perpetrators (Table 3). The largest observed difference was in the relationship between low family SEP and less than good health (OR = 3.60, 95% CI = 2.77–4.67 vs. OR = 1.80, 95% CI = 1.06–3.04), and the smallest difference was in the relationship between low family SEP and high psychosomatic symptoms (OR = 1.27, 95% CI = 1.03–1.56 vs. OR = 1.04, 95% CI = 0.61–1.77).

**Table 3:** Odds ratios (95% confidence interval) of poor health outcomes by two SES measures, adjusted for age, sex, and bullying victimization, stratified by bullying perpetration.

Pathway		Bullying perpetration		
	Total No. of participants	No	Yes	*P-interaction*
		OR (95% CI)	OR (95% CI)	
**Less than good health**				
** Low family SEP**	3096	**3.60 (2.77–4.67)**	**1.80 (1.06–3.04)**	**0.023**
** Low material well-being**	3041	**1.87 (1.51–2.32)**	0.91 (0.51–1.63)	**0.025**
**Low psychosocial well-being**				
** Low family SEP**	3016	**2.79 (2.20–3.53)**	1.50 (0.97–2.34)	**0.017**
** Low material well-being**	2965	**1.60 (1.34–1.91)**	0.96 (0.64–1.45)	**0.020**
**High psychosomatic symptoms**				
** Low family SEP**	2594	**1.27 (1.03–1.56**	1.04 (0.61–1.77)	0.504
** Low material well-being**	2554	0.99 (0.82–1.20)	0.69 (0.42–1.17)	0.186

SEP, socioeconomic position; Bold values denote statistical significance (*P* < 0.05).

To interpret the interaction above, the same model results were reproduced by presenting the relationships between bullying perpetration and poor health outcomes for low- and high-SES adolescents separately (S1 Table). In adolescents from low-SES families, bullying perpetration was significantly or apparently associated with better perceived health status, higher psychosocial well-being, and fewer psychosomatic symptoms. In adolescents from high-SES families, bullying perpetration was associated with increased odds of reporting poor health outcomes. No consistent and conclusive sex differences in the relationship between low-SES measures, bullying perpetration, and poor health outcomes were revealed.

## Discussion

We used data from the HBSC 2013/14 survey to assess the role of bullying in the relationship between SES and health among a nationally representative sample of Armenian adolescents. Contrary to previous research [22–24], bullying perpetration was not associated with low-SES measures in the present study, suggesting a context-depended and possibly multifactorial relationship between SES and bullying perpetration. As a result, adding bullying perpetration to the models did not change the magnitude of the association between low SES and poor health. Therefore, we could not support our first hypothesis that the relationship between low SES and poor health was suppressed by bullying perpetration. However, we found that socioeconomic inequalities in health were weaker among perpetrators, providing evidence for our second hypothesis, that bullying perpetration, as an alternative hierarchy, may compensate for the adverse health effects of low SES in adolescents.

In this study, bullying perpetration was significantly or apparently associated with better perceived health status, better psychosocial well-being, and fewer psychosomatic symptoms in adolescents from low-SES families. Our findings are consistent with the low-status compensation theory [25,26], which suggests that low-SES individuals might use violence to compensate for their low status. Although undesirable, it appears that Armenian adolescents from low-SES families might use bullying perpetration to achieve higher status among their peers, which can make them feel better, whether through improved control and social participation [34] or relieved status anxiety [35]. More research into this (perpetration) and other (perhaps ethically more accepted) coping/compensatory strategies to handle the vicious health-compromising influence of socioeconomic stratification are needed. Qualitative research might provide more insight into the experiences, attitudes, and adaptive strategies of adolescents.

In the present study, we also found that bullying perpetration can be associated with poor health outcomes in adolescents from high-SES families. Research has demonstrated that high-SES children might have a greater degree of achievement pressure, as well as emotional and physical isolation from their parents, which can cause psychosocial maladjustment and internalizing health problems, such as anxiety and depression [36,37], with the potential to create a downward spiral of isolation, maladjustment, and internalizing problems. A growing body of evidence suggests that maladjustment and internalizing issues might predict bullying perpetration [17,38–41], and therefore, it could have increased the risk of perpetrating bullying among high-SES adolescents. Further research is needed to explore this possibility.

There are several limitations to our study. The cross-sectional design makes it difficult to establish a causal relationship because of the possibility of reversed associations between bullying perpetration and poor health in high-SES adolescents. Adolescents might have underreported bullying perpetration because of social desirability bias. Therefore, the association between bullying perpetration and health might be underestimated, perhaps to a greater extent in the high-SES group. Although some of the relationships did not reach statistical significance (*P* < 0.05), our findings were consistent across all health outcomes and SES measures, suggesting robust associations between bullying perpetration and socioeconomic inequalities in health. Approximately 21.9% of adolescents had at least one missing responses related to psychosomatic complaints. However, this might have minimal impact on our results since the proportion of missing values was similar for high and low categories of the SES measures. For other variables, the proportions of respondents with missing data were relatively small (up to 8.6%).

## Conclusions

In conclusion, the relationship between low SES and poor health was weaker in bullying perpetrators, suggesting that bullying perpetration, as an alternative hierarchy, may be looked at as a compensatory, but vicious strategy in the face of the negative health influences of low SES in Armenian adolescents. Context-specific drivers of bullying perpetration are poorly studied in Armenia. Unfortunately, for adolescents from low-SES families, bullying can be a way to achieve a higher position in the peer hierarchy and feel better, whether through improved control and social participation or relieved status anxiety. For high-SES adolescents, on the other hand, social, emotional, or psychological problems may increase the risk of bullying perpetration. Hence, healthier alternatives to bullying perpetration should be encouraged, especially for adolescents from low-SES families. Targeted health interventions might be necessary for high-SES perpetrators. Consequently, bullying prevention activities in Armenia should focus on both low and high-SES adolescents, considering SES-specific pathways and mechanisms. Further studies are needed to identify the contextual factors and the underlying mechanisms for the observed complex relationships between family SES, bullying perpetration, and adolescent health.

## Supporting information

S1 Table Odds ratios (95% confidence interval) of poor health outcomes by bullying perpetration, adjusted for age, sex, and bullying victimization, stratified by family SEP and material well-being. SEP, socioeconomic position; Bold values denote statistical significance (P < 0.05).
(DOCX)
