# Supplementary material for: Pathways to socioeconomic health differences in Armenian adolescents: The role of bullying perpetration
Source: PLoS One. 2022 Jun 3;17(6):e0269451. doi: 10.1371/journal.pone.0269451 (PMC9165766; doi:10.1371/journal.pone.0269451)
Supplement: S1 Table — SEP, socioeconomic position; Bold values denote statistical significance (P < 0.05). (DOCX) [file pone.0269451.s001.docx]

**S1 Table. Odds ratios (95% confidence interval) of poor health outcomes by bullying perpetration, adjusted for age, sex, and bullying victimization,**

**stratified by family SEP and material well-being**

|  | **Family SEP** | | | | | | **Material well-being** | | | | | |
| --- | --- | --- | --- | --- | --- | --- | --- | --- | --- | --- | --- | --- |
|  | **Low** | | **High** | | ***P-interaction*** | | **Low** | | **High** | | ***P-interaction*** | |
|  | **OR (95% CI)** | | **OR (95% CI)** | |  |  | **OR (95% CI)** | | **OR (95% CI)** | |  |  |
| **Less than good health** |  | |  | |  | |  | |  | |  | |
| **Bullying perpetration** | 0.74 (0.47 – 1.16) | | **1.47 (1.01 – 2.15)** | | **0.023** | | 0.78 (0.49 – 1.25) | | **1.61 (1.09 – 2.39)** | | **0.025** | |
| **Low psychosocial well-being** |  | |  | |  | |  | |  | |  | |
| **Bullying perpetration** | **0.60 (0.39 – 0.94)** | | 1.12 (0.83 – 1.50) | | **0.017** | | 0.71 (0.50 – 1.02) | | 1.18 (0.85 – 1.62) | | **0.020** | |
| **High psychosomatic symptoms** |  |  | |  | |  | |  | |  | |  |
| **Bullying perpetration** | **2.13 (1.32 – 3.42)** | | **2.59 (1.85 – 3.63)** | | 0.504 | | **1.95 (1.27 – 2.98)** | | **2.82 (1.99 – 3.99)** | | 0.186 | |
| SEP, socioeconomic position; Bold values denote statistical significance (*P* < 0.05) | | | | | | | | | | | | |
